# Supplementary material for: Screening of Novel Laccase Producers—Isolation and Characterization of Cold-Adapted Laccase from Kabatiella bupleuri G3 Capable of Synthetic Dye Decolorization
Source: Biomolecules. 2021 Jun 2;11(6):828. doi: 10.3390/biom11060828 (PMC8229335; doi:10.3390/biom11060828)
Supplement: Supplementary file 1 [file biomolecules-11-00828-s001.zip › biomolecules-1232677-supplementary.pdf]

Screening of Novel Laccase Producers—Isolation and Characterization of Cold-Adapted Laccase from *Kabatiella bupleuri* G3 Capable of Synthetic Dye Decolorization

Katarzyna M. Wiśniewska, Aleksandra Twarda-Clapa and Aneta M. Białkowska *

Institute of Molecular and Industrial Biotechnology, Lodz University of Technology, Stefanowskiego 4/10, 90-924 Łódź, Poland * Correspondence: aneta.bialkowska@p.lodz.pl

SUPPLEMENTARY MATERIALS

Contents

Figure S1 2

Figure S2 3

Table S1 4

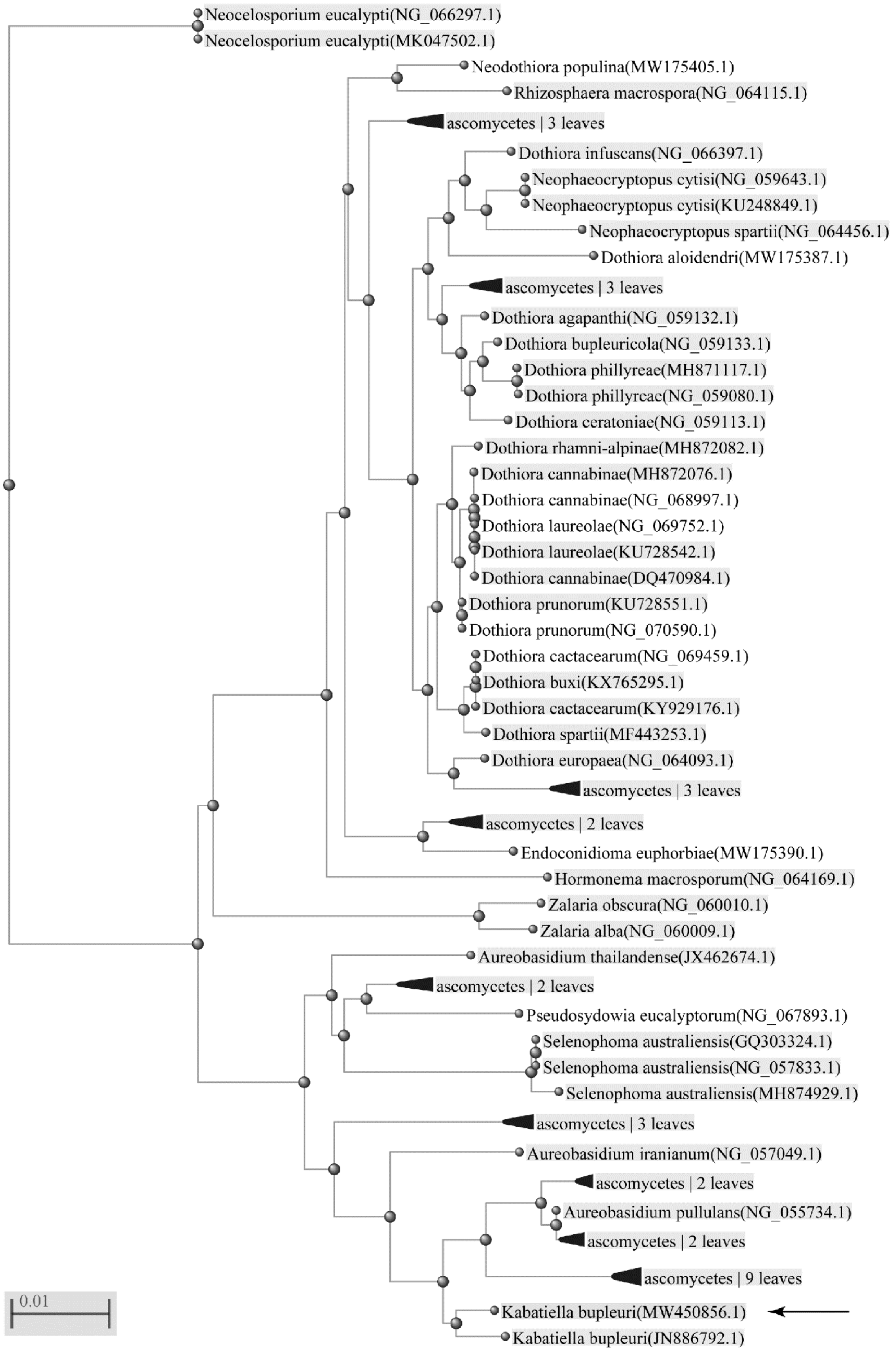

Figure S1. Phylogenetic similarity of strain G3 IBMiPto other yeast strains based on the analysis of D1/D2 sequences. Black arrow indicates the sequences from G3 IBMiP; the remaining sequences are described by species name and GenBank accession number.

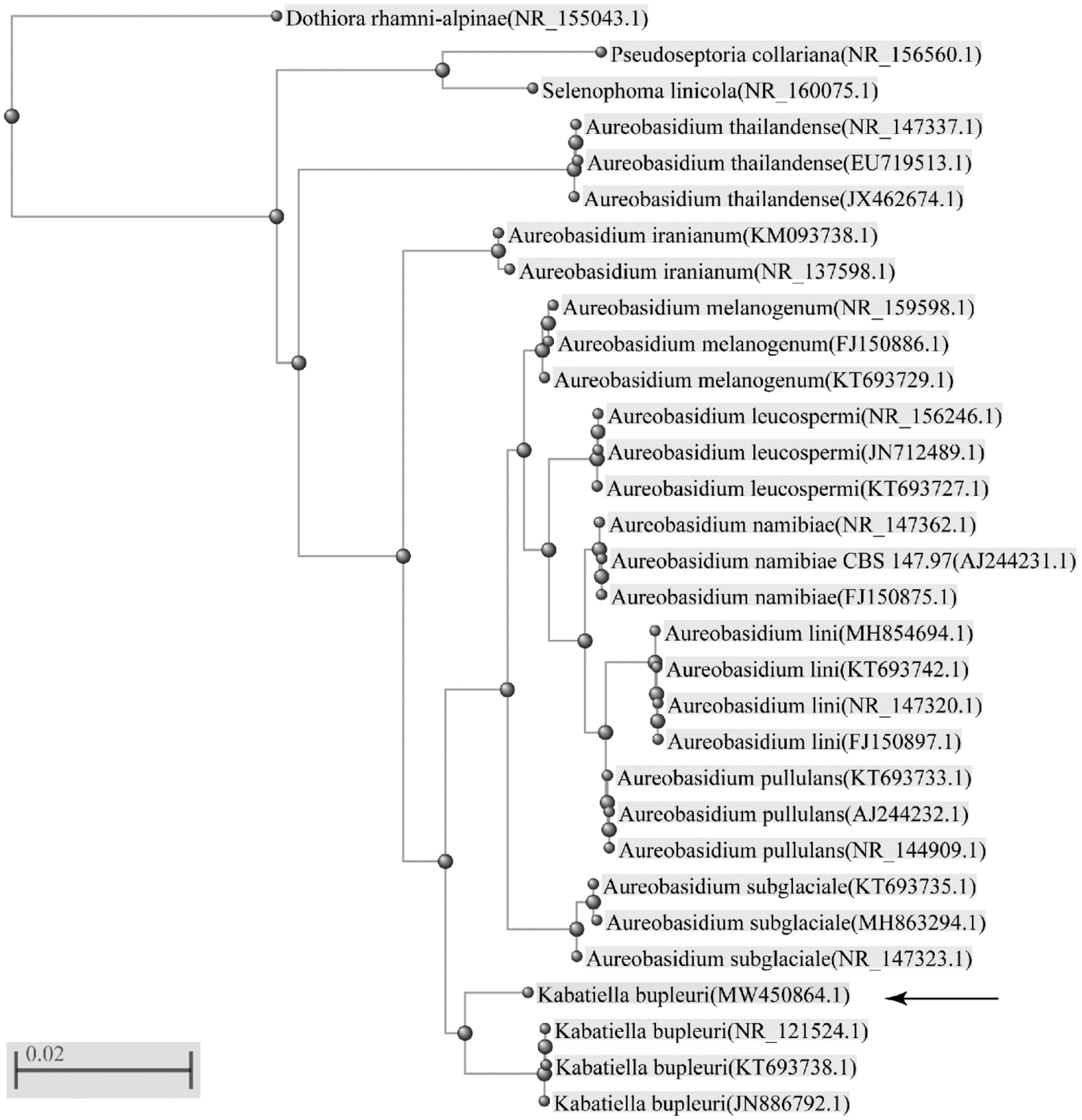

Figure S2. Phylogenetic similarity of strain G3 IBMiP to other yeast strains based on the analysis of ITS1&ITS2 sequences. Black arrow indicates the sequences from G3 IBMiP; the remaining sequences are described by species name and GenBank accession number

Table S1. Influence of inducers (at the optimal concentration) on extracellular laccase activity (U/L) during 30 days culture.

	10 days	15 days	20 days	25 days	30 days
**control**	9.74 ± 0.99	19.59 ± 0.95	50.71 ± 2.03	44.48 ± 2.04	34.80 ± 1.67
**30 µM 2,5-xylidine**	9.95 ± 1.06	15.99 ± 0.44	38.31± 0.39	33.94 ± 0.84	26.91 ± 0.89
**1 mM veratryl alcohol**	11.41 ± 0.82	14.84 ± 0.94	51.11 ± 1.07	49.47 ± 0.84	37.61 ± 0.84
**1 mM vanillin**	9.43 ± 0.82	16.92 ± 0.98	46.33 ± 0.07	43.81 ± 0.54	34.65 ± 0.88
**0.26 µM guaiacol**	4.68 ± 0.71	20.08 ± 0.57	40.09 ± 0.54	35.46 ± 0.94	25.72 ± 0.99
**0.11 µM syringaldazine**	7.46 ± 0.79	14.89 ± 0.96	56.41 ± 1.14	51.06 ± 0.67	40.11 ± 0.74
**1.8 µg/ml ethidium bromide**	9.25 ± 0.77	20.52 ± 0.80	60.05 ± 1.55	53.53 ± 0.47	40.51 ± 1.32
**1 mM catechol**	9.61 ± 0.76	15.36 ± 0.97	58.40 ± 1.57	46.78 ± 0.70	36.98 ± 0.43
**1 mM Tween 20**	0.00 ± 0.00	0.09 ± 0.04	4.67 ± 0.39	5.36 ± 0.40	5.88 ± 0.47
**1 mM Tween 80**	2.25 ± 0.71	30.61 ± 1.71	70.72 ± 1.00	64.63 ± 0.81	49.29 ± 1.38
**1 mM ABTS**	3.02 ± 0.71	3.17 ± 0.30	4.13 ± 0.38	5.84 ± 0.86	7.96 ± 0.63
